# Process evaluation of implementation fidelity of the integrated chronic disease management model in two districts, South Africa

**DOI:** 10.1186/s12913-019-4785-7

**Published:** 2019-12-16

**Authors:** Limakatso Lebina, Olufunke Alaba, Ashley Ringane, Khuthadzo Hlongwane, Pogiso Pule, Tolu Oni, Mary Kawonga

**Affiliations:** 1 Perinatal HIV Research Unit (PHRU), SA MRC Soweto Matlosana Collaborating Centre for HIV/AIDS and TB, Faculty of Health Sciences, University of the Witwatersrand, Johannesburg, South Africa; 2 Division of Public Health Medicine, School of Public Health and Family Medicine, University of Cape Town, Cape Town, South Africa; 3 Health Economics Unit, School of Public Health and Family Medicine, University of Cape Town, Cape Town, South Africa; 4 MRC Epidemiology Unit, University of Cambridge, Cambridge, UK; 5 Department of Community Health, School of Public Health, Faculty of Health Sciences, University of the Witwatersrand, Johannesburg, South Africa

**Keywords:** Intervention adherence, ICDM model, Chronic care model, Implementation research, Value stream mapping

## Abstract

**Background:**

The Integrated Chronic Disease Management (ICDM) model has been implemented in South Africa to enhance quality of clinical services in Primary Healthcare (PHC) clinics in a context of a high prevalence of chronic conditions and multi-morbidity. This study aimed to assess the implementation fidelity (adherence to guidelines) of the ICDM model.

**Methods:**

A cross-sectional study in 16 PHC clinics in two health districts in South Africa: Dr. Kenneth Kaunda (DKK) and West Rand (WR). A fidelity assessment tool with 89 activities and maximum score of 158 was developed from the four interrelated ICDM model components: facility re-organization, clinical supportive management, assisted self-management and strengthening of support systems. Value stream mapping of patient flow was conducted to analyse waiting time and identify operational inefficiencies. ICDM items were scored based on structured observations, facility document reviews and structured questionnaires completed by healthcare workers. Fidelity scores were summarized using medians and proportions and compared by facilities and districts using Chi-Square and Kruskal Wallis test.

**Results:**

The monthly patient headcount over a six-month period in these 16 PHC clinics was a median of 2430 (IQR: 1685–2942) individuals over 20 years. The DKK district had more newly diagnosed TB patients per month [median 5.5 (IQR: 4.00–9.33) vs 2.0 (IQR: 1.67–2.92)], and fewer medical officers per clinic [median 1 (IQR: 1–1) vs 3.5 (IQR:2–4.5)] compared to WR district. The median fidelity scores in both districts for facility re-organization, clinical supportive management, assisted self-management and strengthening of support systems were 78% [29/37, IQR: 27–31)]; 77% [30/39 (IQR: 27–34)]; 77% [30/39 (IQR: 28–34)]; and 80% [35/44 (IQR: 30–37)], respectively. The overall median implementation fidelity of the ICDM model was 79% (125/158, IQR, 117–132); WR was 80% (126/158, IQR, 123–132) while DKK was 74% (117/158, IQR, 106–130), *p* = 0.1409. The lowest clinic fidelity score was 66% (104/158), while the highest was 86% (136/158). A patient flow analysis showed long (2–5 h) waiting times and one stream of care for acute and chronic services.

**Conclusion:**

There was some variability of scores on components of the ICDM model by PHC clinics. More research is needed on contextual adaptations of the model.

## Background

Delivering integrated, patient-centred health services is a global public health priority [1]. One of the recommended strategies of the World Health Organization (WHO) to improve the delivery of integrated chronic care at primary care level is the Innovative Care for Chronic Conditions framework, which reduces fragmentation of care and supports partnerships with communities and families in disease management [2]. Based on this recommendation, many countries have implemented various models of integrated care for chronic conditions, including community-based programmes with repeat collection of medication [3–5], nurse-managed programmes [5] and integrated mental health, diabetes and cardiovascular disease services [6]. In South Africa, the Integrated Chronic Disease Management (ICDM) model was adopted as a national programme for implementation at primary care level. The goal was to reduce fragmentation of care for people living with multiple chronic conditions, to improve efficiency and the satisfaction experience of healthcare workers and patients, and ultimately clinical outcomes [7]. This was also in response to the high quadruple burden of communicable, non-communicable, injury-related and perinatal diseases and associated multi-morbidity [8–11].

### The ICDM model

The ICDM model is an approach to managed care that was developed based on the principles of the chronic care management model and the Innovative Care for Chronic conditions (ICCC) [2, 12]. A pilot phase of introducing the model in PHC clinics in South Africa started in 2011 [13]. The ICDM model provides technical interventions on how to strengthen health services for patients with chronic multi-morbidity through four interrelated components of action points. These components are as follows [7]:

- *Facility re-organization* to strengthen administration, infrastructure, human resource and dispensing of medication for operational efficiency;
- *Clinical supportive management* to enhance quality care using appropriate clinical guidelines with the assistance of the district clinical specialist team;
- *Assisted self-management* which entails empowering patients to take responsibility for their disease control and providing community-based point-of-care testing and medication delivery by outreach teams; and
- *Strengthening of support systems* through partnerships with external structures, equipment, medicine and information management [7].

The ICDM model’s priority standards are designed to align with the national core quality standards for PHC facilities, which include patient safety and infection control, improving values and attitudes of staff, improving waiting times and cleanliness, and ensuring availability of medicines and supplies [7]. The chronic diseases that are included in the ICDM model are non-communicable [mental health, epilepsy, asthma, hypertension, diabetes and chronic obstructive pulmonary disease (COPD)] and communicable diseases [HIV/AIDS and all forms of tuberculosis (TB)] [7]. Continuum of care is supported at facility, community and population level under the ICDM model [7].

The ICDM programme is an integral part of the PHC re-engineering initiative [7], a major health system strengthening focus of which is the South African Department of Health’s (DOH) efforts to strengthen their focus as it works towards achieving universal health coverage through a national health insurance plan [14–16]. This includes the ongoing ideal clinic realisation and maintenance (ICRM) programme that was commenced in 2013 [17]. The ICRM programme is a comprehensive systematic process of improving and maintaining PHC facilities’ conformance to national standards on functional infrastructure and equipment, adequate personnel and medicines and supplies, good administrative processes and the use of applicable protocols and guidelines in diseases management [17]. The Integrated Clinical Services Manual (ICSM) was included in the ICRM programme to extend the application of the ICDM model components to acute, preventative and health support services as part of scaling-out [18]. Chronic diseases has been included as one of the streams of the ICSM [18]. A scaling-out of interventions (delivery in new systems/populations) or scaling-up within the same context implies that the original core elements are maintained to achieve expected outcomes [19]. However, contextual adaptations to the intervention while maintaining the core components in the scale-up and scale-out could also be regarded as propensity towards adherence [20]. Studies on the effectiveness of the ICDM model have shown its contribution to improvements in patients records management through administrative re-organization and improved clinical outcomes through clinical supportive management and assisted self-management for patients on antiretroviral medication [21, 22]. However, some of the expected benefits have not been achieved [23]. It is not clear whether this limited success indicates low effectiveness of the model or low implementation effectiveness.

The field of implementation science provides approaches for assessing implementation effectiveness [24, 25]. Implementation research assists in assessing whether the failure of an intervention to achieve its outcomes is as a result of intervention failure or implementation failure [24]. Implementation effectiveness or success can be determined by measuring implementation outcomes such as fidelity (the extent to which the ICDM model is implemented according to the planned prescribed activities) or other outcomes (including acceptability, adoption, reach, implementation costs and sustainability) that serve as intermediate indicators of intervention or innovation effectiveness [24, 26]. Fidelity of implementation – the extent to which delivery of an intervention or programme follows the original design – affects how well the intervention or programme achieves its expected outcomes [27, 28]. Fidelity is also referred to as adherence to intervention guidelines [28]. As conceptualised by Carroll, the degree of adherence to the implementation plan or guidelines can be influenced by moderating factors like intervention complexity, strategies to support implementation, quality of delivery and participant responsiveness [28]. Planned or accidental adaptations in implementing interventions could also be viewed as strategies to enhance feasibility, reach, adoption, and acceptability of the intervention in a specific context [29, 30]. However, adaptation could affect the fidelity and effectiveness of the intervention, especially if its core components have been removed or modified [19, 29, 30]. Therefore, there is a constant tension between fidelity and modifying interventions to be suitable for a particular context [29, 30]. Since the ICDM model development was an adaptation of the ICCC for the South African health context [7], it would be expected that it would be implemented with minimal adaptations and high fidelity, but this has not been evaluated. Moreover, in a decentralized health system, like South Africa, sub-national levels (provinces and districts) may further adapt innovations for a better fit with their contexts [31]. However, whether and the extent to which such further adaptations have been done has not been evaluated.

In South Africa, following the pilot and scale-up of the ICDM model [32], there is a dearth of studies on the implementation fidelity of the ICDM model. This study assesses the implementation fidelity, which we define as adherence to the prescribed activities in the ICDM model as outlined in the implementation manual [7]. This study aims to evaluate the implementation fidelity of the ICDM model in two health districts in South Africa. The lessons learned on assessing fidelity of the ICDM model could be applied to the ICSM in the context of the ICRM programme. Assessing the ICDM model implementation fidelity will identify areas that need strengthening to promote the sustainability of the model’s principles.

## Methods

This was a cross-sectional study conducted between August 2018 to March 2019 in two health districts in South Africa. It is a sub-study of a larger study that assessed the fidelity of implementation, its contextual determinants and the costs of implementing the ICDM model [33].

### Study setting

The South African health system is divided into 52 health districts across nine provinces, with health service administration decentralized to district health management teams [16, 34]. Most of the population is uninsured (82%) and utilizes state facilities where most healthcare services are free or provided at a low user fee [14, 35, 36]. Nurse-driven primary care services in each district are provided at PHC clinics (8-h service) and community health centres (24-h service) that provide preventative and curative (acute and chronic) services. As part of the PHC re-engineering framework, each clinic should have ward-based outreach teams (WBOTs) of community healthcare workers (CHCW) providing home- and community-based health services [17, 37]. Each health districts is required to have a District Clinical Specialist team (DSCT) consisting of specialist nurses and doctors that provide supportive supervision and clinical governance [17, 38]. The ICDM model activities for the WBOTs and CHCW include adherence support, home-based care and community campaigns, while the DCST activities include mentoring, training and clinical audits [7, 18].

The ICDM model was piloted from 2011 in three health districts: West Rand (WR) in Gauteng, Bushbuckridge in Mpumalanga and Dr. Kenneth Kaunda (DKK) in North West [13]. Two (WR and DKK) of these health districts were included in this study. Both the WR and DKK health districts are divided into four sub-districts and have similar population sizes, 810,000 in WR and 715,000 in DKK [39]. There are four community health centres and 39 PHC clinics in WR, while DKK has nine community health centres and 27 PHC clinics. Although the literacy rate is slightly higher in WR (98% vs 90%), employment is higher (75% vs 71%) in DKK [40]. In WR, the proportion of informal housing is 19%, while in DKK it is 21% [40]. In Gauteng, more people (34%) are considered to be obese or overweight compared to the 16% in North West [41]. There is also a high prevalence of hypertension (36 and 39%) [39] and diabetes (8 and 13%) [41] in WR and DKK, in that order. TB incidence is higher (696 vs 440 per 100,000) in DKK [39], and the human immune deficiency syndrome (HIV) prevalence in antenatal women is 28% in both provinces [42].

### Study sample

There were eight ICDM model pilot clinics in DKK and 12 in WR that were considered for inclusion in this study. The ICDM model pilot clinics were selected for inclusion based on the number of patients that receive health services per month (headcounts) to ensure that clinics had comparable patient case-loads. Four clinics from the WR district were excluded as the patient load in those clinics was much higher compared to DKK clinics. A total of 16 (eight per district) that were selected were functional without major interruptions (closures, renovations) in the 2 years preceding participation in the study. Six to eight healthcare workers (administrators, nurses, pharmacists assistants, facility managers, ICDM champions) were interviewed (for completion of the structured questionnaire) or observed in each facility.

### Data collection and measurement

The study aimed to collect data on the characteristics of the clinics, fidelity scoring on ICDM model activities and examination of patient flow against guidelines. The data collected on clinic characteristics included a facility headcount, caseloads for some (HIV/AIDS, TB, diabetes and hypertension), ICDM model conditions and number of different categories of personnel based on district health information system reports. The monthly patient data (headcounts, caseloads) were collected for a period of 6 months.

To measure fidelity (adherence to ICDM model activities), we first developed fidelity criteria based on the ICDM model manual [7] with a focus on the recommended activities, the recommended reporting tools for the ICDM model and ICRM programme assessment tools. Since no previous studies have assessed the implementation fidelity of the ICDM model, we developed an ICDM model implementation fidelity assessment tool for this study. Our ICDM fidelity assessment tool was designed to measure the extent to which activities under each of the four major components of the ICDM model (facility re-organization, clinical supportive management, assisted self-support and strengthening of support systems) [7] were implemented according to the ICDM model design. Each of the four ICDM model components has various activities that must be implemented to achieve the aims of the ICDM programme [7]. These activities were used to form the basis of the variables to be measured on the implementation fidelity assessment tool. Our fidelity tool was therefore a checklist of variables (activities) under each component. They were scored following similar principles as other chronic diseases management model evaluation studies [43]. As the ICDM model is prescriptive on how activities should be implemented to support integrated care for chronic patients, we posited that failure to implement the recommended activities was regarded as low fidelity.

**Fig. 1:**
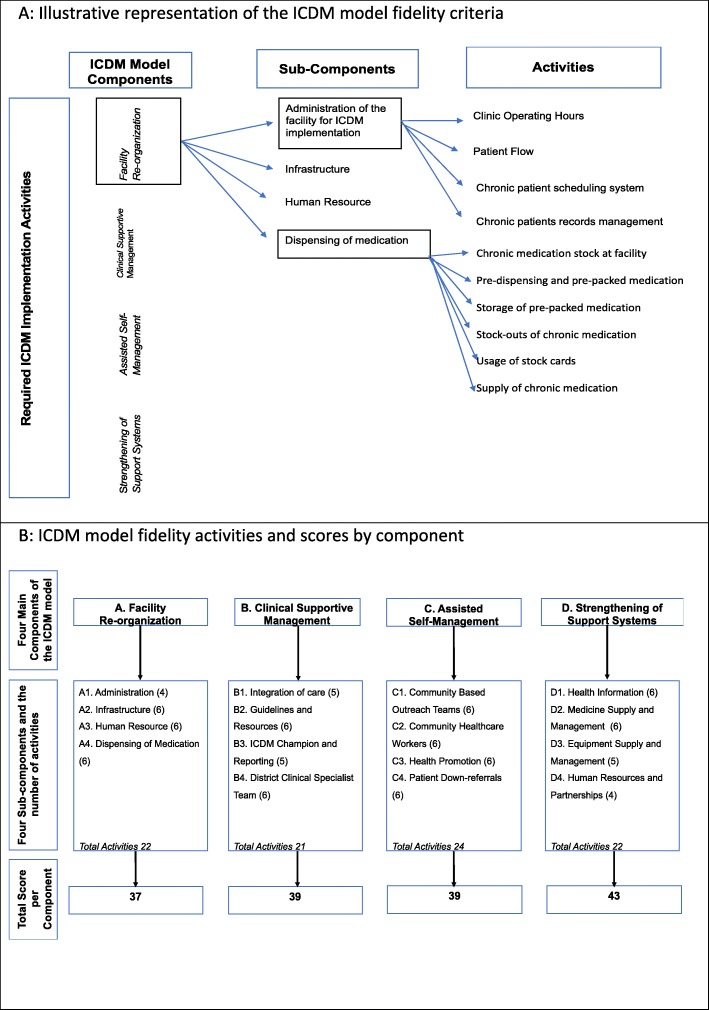
Illustrative representation of the Integrated Chronic Disease Management Model Fidelity Criteria, and the scores by component. **a**: Illustrative representation of the ICDM model fidelity criteria. **b**: ICDM model fidelity activities and scores by component

Each of the four components of the ICDM model comprises four sub-components and each sub-component comprises of four to six activities as outlined in the ICDM model manual (Fig. 1a) [7]. A total of 89 activities or items (facility re-organization 22; clinical supportive management 21; assisted self-management 24, and strengthening of support systems 22) were thus measured in the fidelity assessment tool (Additional file 1). The activities were each scored on a scale, with activity scores ranging from 0 (not implemented at all) to 4 (fully implemented as planned – adherent). The activity (item) scores varied depending on the details of the activity. For example, the scores for the activity “pre-dispensing and packing of chronic medication 2–3 days prior to visit” were zero if not implemented, and a maximum score of one if implemented, whereas the score for the activity “building” ranged from 0 (needs major repairs) to 2 (no major repairs needed and floors and walls clean). The total maximum possible fidelity score was 158 per facility (facility re-organization 37; clinical supportive management 39; assisted self-management 39 and strengthening of support systems 43; Fig. 1b).

The ICDM model activities fidelity assessment tool was piloted in four clinics and thereafter revised for clarity and consistency in scoring. Fidelity scoring for the 89 activities was performed through structured observations for such activities like the availability of guidelines and resources, infrastructure maintenance and medicine supply and management. For other items of the ICDM model where observation would be challenging, such as training of healthcare workers, support provided by the DCST and the activities of the WBOT and CHCW in the community were scoring was based on structured questionnaires with healthcare workers. Staff members involved in the implementation of the ICDM model’s various components were selected for further data collection using structured questionnaires interviews. The interviews in this study were structured with the aim of assessing how certain activities of the ICDM model that could not be assessed by record review or observations had been performed in the facilities. In addition, all available documents relevant to ICDM model implementation at each facility (human resource and administration files, medicine, and equipment management documents) were reviewed to score the scheduling system and dispensing of medication, among others. Data collection was done over a period of 8 months with multiple visits to the health facilities on different days of the week and times of the month to gather data on a variety of activities. The research team was trained on the protocol and the data collection tools. This team then conducted the pilot and refining of the fidelity assessment tool prior to data collection and fidelity scoring in all the PHC facilities for consistency.

To further examine adherence to the ICDM model guidelines and cross verification of the fidelity scores, we also conducted a patient flow analysis using value stream mapping [44] to ascertain if the processes followed are aligned with the ICDM model guidelines. Patient flow analysis following the value stream mapping was done in four clinics, one with the highest and one with the lowest fidelity scores per district, but with comparable average monthly PHC headcounts. The data collected on the structured observations of patient flow included where and who provided various services for patients with chronic diseases, time for the service, the waiting times and areas of inefficiency. An average of 15 patients with a chronic disease per facility were observed from entry into facility till exit.

#### Data management and analysis

The data collected on the paper-based ICDM model fidelity assessment tool in the field were captured into a REDCap electronic database [45]. Each facility was allocated a number for study identification and no identifiers were included in the password-protected REDCap database. As part of data cleaning, the data were checked for apparent discrepancies, missing variables and incorrect data. The data were exported into Excel and Statistical Analysis Software (SAS) Enterprise Guide 7.1 for analysis [46].

Descriptive statistics (frequency, median, interquartile ranges, percentages) were used to summarize the data on general clinic characteristics, like personnel, the number of chronic patients, and the services offered. Fidelity item scores were summed per component to give four ICDM model component fidelity scores per facility. An overall ICDM model implementation fidelity score was determined per facility by summing the four sub-component scores. The ICDM model implementation fidelity scores were summarized using descriptive statistics (medians and IQR, and converting scores to proportions) and compared across facilities and districts using the Kruskal Wallis and Chi-square tests. In the South African DOH ICRM programme, facilities are scored for ideal clinic status as silver (70–79%), gold (80–89%) and platinum (90–100%), based on assessment on 208 elements, categorized into 10 components covering administration, clinical services provision and health outcomes [17]. A score below 70% or failure to achieve a minimum percentage in the vital elements is rated as not having achieved ideal clinic status [17]. Although the fidelity assessment of the ICDM model in this study did not encompass all the elements of the ideal clinic, we used similar categories (silver, gold and platinum) in interpreting the fidelity scores because there are no existing norms regarding what constitutes high fidelity of implementation for a chronic care model.

### Ethical considerations

This study was approved by the University of the Witwatersrand (Ref: R14/49) and University of Cape Town’s (Ref: 127/2018) Human Research ethics committees. The Gauteng and the North West provincial departments of health also gave their approval.

## Results

### Characteristics of the clinics

All the PHC facilities provided nurse-driven curative and preventative health services and had been implementing the ICDM model since 2011. As shown in Table 1, the 16 PHC facilities that were included in the study sample provided health services to a varied number of patients every month, with a median of 2430 (IQR: 1685–2942) patients aged > 20 years accessing care per facility per month. However, the PHC monthly headcount varied ranging from an average of 857 to 4946 patients seeking health services. When comparing the two districts, the DKK district had significantly (*p* = 0.0117) more [median 5.5 (IQR: 4.00–9.33) vs 2.0 (IQR: 1.67–2.92)] patients ≥5 years diagnosed with TB monthly. The WR district had significantly more medical officers [3.5 (IQR: 2–4.5) vs 1.0 (IQR: 1.0–1)] and enrolled nurses [3.5 (IQR: 3–5.5) vs 0.0 (IQR: 0.0–1.5)] per facility. All facilities had access to at least one medical officer, and each facility had a facility manager. Six facilities did not have a pharmacist assistant.

**Table 1:** Characteristics of the Primary Health Care Clinics by health district

Variables	WR District Median (IQR)	DKK District Median (IQR)	*P*-Value
Primary healthcare headcount per month per facility	3361 (2430–4173)	3690 (2083–3953)	0.9164
Primary healthcare headcount of patients > 20 years old per month per facility	2277 (1685–3098)	2626(1584–2942)	0.8336
Number of HIV/AIDS Adult remaining on ART per facility	1525 (1070–1816)	1572 (624–2114)	0.9164
Number of new Tuberculosis cases (≥ 5 years old) per month per facility	2 (1.67–2.92)	5.5 (4.00–9.33)	0.0117
Number of new Diabetic patients (≥ 40 years) per month per facility	8.83 (5.08–10.5)	9.67 (4.00–13.2)	0.6982
Number of diabetic patients case load per month	66.3 (43.5–89.3)	67.8 (36.1–91.4)	0.7527
Number of hypertensive patients case load (*visits*) per month per facility	286 (252–395)	252 (233–405)	0.4622
Number of Professional Nurses per facility	7.0 (5.5–9.0)	5.5 (5.0–11)	0.7105
Number of Enrolled Nurses per facility	3.5 (3.0–5.5)	0.00 (0–1.5)	0.0053
Number of Medical Officers per facility	3.5 (2.0–4.5)	1.0 (1.00–1)	0.0012
Number of counselors per facility	3.0 (3.0–3)	4.5 (2.5–6.5)	0.1685
Ratio of Nurses to PHC monthly headcount per facility	305 (224–358)	408 (303–738)	0.1415
Ratio of Medical Officers PHC monthly headcount per facility	1137 (901–1410)	3690 (2083–3953)	0.0087

### ICDM model implementation fidelity

The overall (summation of all component scores) ICDM implementation fidelity score per facility ranged from 68% (108/158) to 86% (136/158). The overall fidelity score was 70 to 79% (silver status) in six clinics, ≥ 80% in eight clinics (gold status) and below 70% (not achieved) in two clinics. The median ICDM implementation fidelity score was 125/158 (IQR: 119–131; 79%) across both health districts. Strengthening of support systems and facility re-organization were the highest (silver) scoring ICDM model components with a score of 79%, while assisted self-management score was 78% and the clinical supportive management was the lowest with 76%. The Cronbach’s alpha (internal consistency of the activities fidelity scoring questions) on clinical supportive management and strengthening of support systems was 0.69, while for facility re-organization and assisted self-management support it was 0.53 and 0.56 respectively. A calculated score of the Cronbach’s alpha that is closer to one indicates a high level of inter-relatedness of the items within a scale [47].

The ICDM model’s four component activity scores (added and individually) were also compared between clinics and health districts.

#### Facility re-organization

**Fig. 2:**
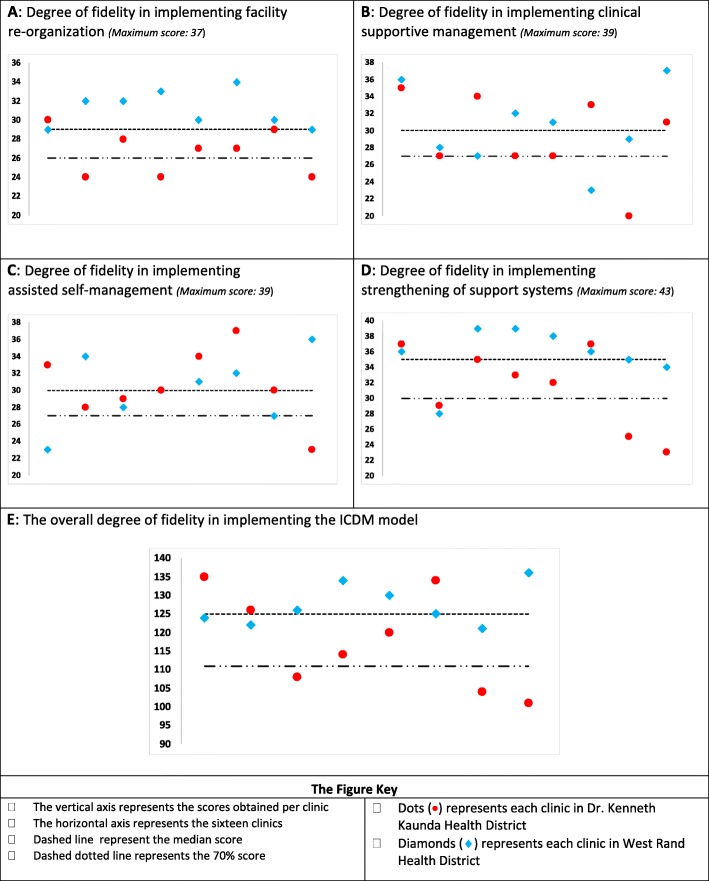
Graphic representation of the degree of fidelity for the four major components of the ICDM model and overall in the implementation of ICDM model in sixteen clinics. **a**: Degree of fidelity in implementing facility re-organization *(Maximum score: 37*). **b**: Degree of fidelity in implementing clinical supportive management *(Maximum score: 39*). **c**: Degree of fidelity in implementing assisted self-management *(Maximum score: 39*). **d**: Degree of fidelity in implementing strengthening of support systems *(Maximum score: 43*). **e**: The overall degree of fidelity in implementing the ICDM model

The overall score for facility re-organization was silver status (79%; 462/584), and the lowest scoring clinic had a score of 65% (24/37), while the highest clinic score was 92% (34/37). The median facility re-organization score was 29/37 (IQR: 27–31; 78%) (Fig. 2). The scheduling of appointments and different streams of care were the least implemented. Nine clinics scored below 75% (6/8) on dispensing medication and one clinic could not be assessed as it had pharmacy support from a hospital-based pharmacy and medication storage and dispensing was not done at the clinic. Half of the clinics obtained scores of 75% or higher on administrative procedures, infrastructure, personnel training and allocation. Medication is stored in the consulting rooms in most (15/16) of the facilities to improve efficiency according to the ICDM guidelines. However, the medicine supply and management principles (e.g. stock cards, temperature monitoring) were only applied to the medication storage room and not in the consulting rooms where some of the medication is being stored.

#### Clinical supportive management

The clinical supportive management overall score across the two districts was silver status (76%; 477/624). The lowest score obtained per facility was 51% (20/39), and the highest score was 95% (37/39). The median clinical supportive management score was 30/39 (IQR: 27–34; 77%) (Fig. 2). Only six clinics had the appropriate clinical guidelines available and accessible. Three clinics did not have access to a DSCT. Half of the clinics had a score of 31/39 (80%) or more on clinical supportive management (Fig. 2). The scores were high due to the high scores on the activities relating to integration (space, time, healthcare worker, medical records) of care and monitoring and reporting on ICDM implementation. Although all the seven chronic conditions recommended for inclusion into one stream of care, TB services had a separate stream (separate medical records, healthcare worker, and consulting rooms).

#### Assisted self-management

The overall score for assisted self-management was also silver status (78%; 485/624). The minimum facility score was 59% (23/39) and the maximum facility score was 95% (37/39). The median assisted self-management score was 30/39 (IQR: 28–34; 77%). Nine clinics scored ≤63% on health promotion as they did not have regular health promotion talks or chronic diseases’ resource material for patients. The score per facility for about two thirds (10/16) of the clinics was above 30/39 (76%). Almost all (15/16) of the clinics had functional WBOTs and were therefore able to implement down referrals and other pick-up points for chronic medication collection in the community.

#### Strengthening of support systems

The overall score for strengthening of support systems across the two districts was silver status (79%;536/675). The lowest score obtained per facility was 53% (23/43), while the highest score was 91% (39/43). The median strengthening of support systems score was 35/43 (IQR: 30–37; 81%). The lowest (23/43; 53%) scoring clinic failed on health information as it did not use the appropriate data collection tools. The least implemented activities were the school health team and equipment supply and management. Ten (10/15; 67%) clinics had a stock visibility system and still used the manual stock cards for medication stock levels monitoring. Most (11/16) of the clinics scored ≥75% (33/43) on strengthening of support systems.

**Fig. 3:**
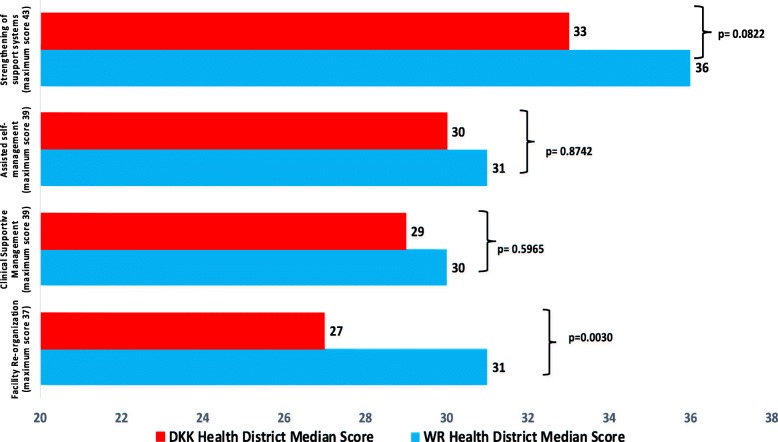
Comparison of the fidelity scores for the four major components of the ICDM model by health district

Although the median overall fidelity score for WR was higher than for DKK, the difference was not statistically significant (126, IQR: 123–132 vs. 117, IQR: 106–130; *p* = 0.1409). The median facility re-organization fidelity score was significantly higher in the WR than in the DKK (31 vs 27/37; *p* = 0.0030) health district (Fig. 3). There was no significant difference in the supportive management, assisted self-managed and strengthening support systems fidelity scores between the two districts (Fig. 3), even though the WR district median scores for all three of those components were higher than those of the DKK district.

### Value stream mapping of patient flow

#### Administration

There was poor adherence to the ICDM recommendation to have files pre-retrieved the day before for booked patients, except in one clinic. Administrators only retrieved the medical records for patients that have submitted a clinic card or identity document. The administrator would then update the PHC paper-based and electronic registers before patients move to the vital signs station. All the facilities had a separate stream of care for mother-and-child (preventative and promotive) and TB services. However, there was only one stream for chronic and acute care services. In some cases, the next appointment for review was scheduled for 6 months after blood tests, and this could delay issuing of results and taking the necessary clinical actions depending on the received results, like the change of medication, adherence counselling etc. Observed inefficiency was on excess personnel motion as nurses did not have all the required resources in one consultation room and completion of multiple similar documents like a script in the file and for central chronic medicines dispensing and distribution (CCMDD).

#### Dispensing of medication

Although the clinics did not pre-pack medication, a 2 months’ supply of medication was issued at each visit. Repeat medication collection followed the spaced and fast-line appointment. However, the collection was from the same consulting room or the pharmacy assistant. CCMDD was accessible at three clinics. The allocation of PHC nurses to CCMDD or pharmacy management reduced the number of nurses available to provide primary health care consultations.

#### Waiting and service times

**Fig. 4:**
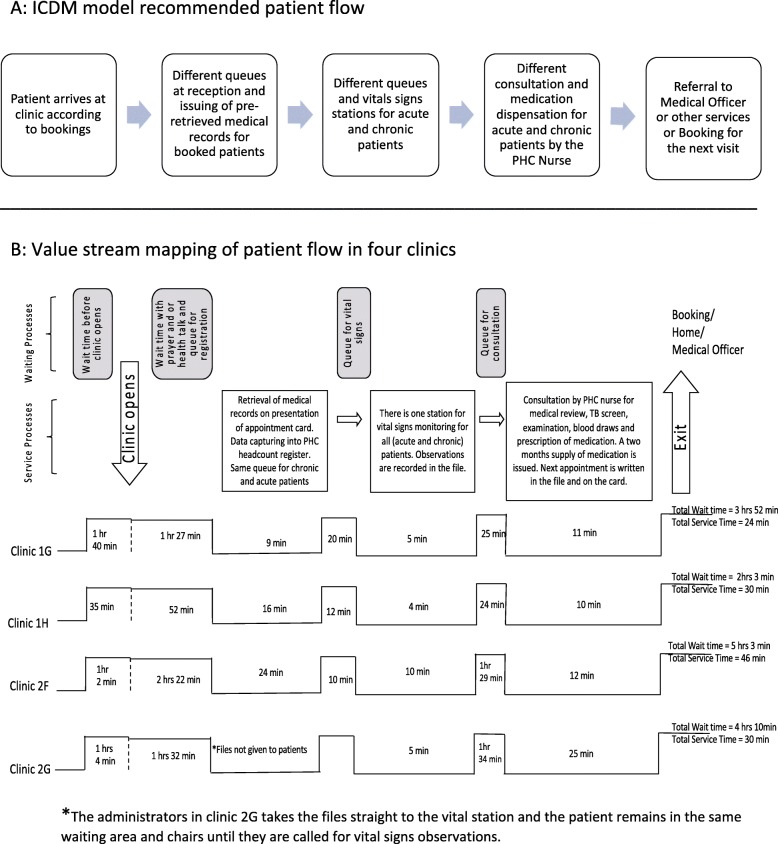
ICDM model recommended patient flow and value stream mapping of patient flow in four clinics. **a**: ICDM model recommended patient flow. **b**: Value stream mapping of patient flow in four clinics

On average, patients spent a total of 4 h 20 min (minimum: 2 h 33 min. and maximum: 5 h 49 min.) at the facility to access health services (Fig. 4). Most (87%; 3 h; 47 min) of the time was spent waiting for care and 13% (33 min) for receiving services. The majority (70%; 43/61) of the observed patients spent 3 h or more at the PHC facility. At the clinic that had the shortest waiting time, patients arrived at different times throughout the day, and the average wait prior to retrieval of medical records was 1 h 27 min, compared to 3 h in the other clinics.

## Discussion

Based on the structured questionnaires, objective observations and facility record reviews, the implementation fidelity of the ICDM model was 79% in the two health districts, with some variability between PHC facilities. Fourteen PHC clinics had a an ICDM implementation fidelity score of ≥70% on implementation of the ICDM model. The clinics in the WR health district had higher fidelity scores compared to those in DKK for all four ICDM components, but the differences were not statistically significant except for the for the facility re-organization component. Scheduling of appointments, pre-retrieval of medical records, different streams of care, and equipment supply and management were the least implemented ICDM model activities. In addition, access to clinical guidelines and support by the DCST was inadequate at some of the clinics. There were high fidelity scores on integration of care, infrastructure, functional WBOTS and medicine supply and management. Waiting time for services was over 3 h, mostly at the medical records retrieval station. Observed unintended consequences of implementing the ICDM model guidelines included reduced personnel for patient care when nurses are allocated to CCMDD or pharmacy, and medication management in consulting rooms. The findings from this study give valuable information on the level of fidelity in the implementation of the ICDM model at a time that the South African DOH is focusing on primary healthcare revitalization in preparation for the national health insurance [14–16].

Although the scoring for the ICDM model fidelity scoring did not contain all the components included in the ICRM programme, applying the ICRM scale [silver (70–79%), gold (80–89%) and platinum (90–100%)] to our study would imply that 12.5% (2/16) clinics in this study did not achieve ideal clinic status on chronic disease health services. Silver status (70–79%) was obtained by 37.5% (6/16) of the assessed clinics, while 50% (8/16) achieved gold status (80–89% on chronic services). No clinic score fell into the platinum category in this study. The higher number of clinics scoring silver and gold status compared to not achieved ideal clinic status (12.5% vs 87.5%) could be indicative of broad improvements in clinic functioning (infrastructure, personnel and supply chain management) under the ICRM programme [17]. In a peer-peer review conducted in 2016 under the ICRM scale-up process, the number of clinics scoring over 70% (achieved ideal clinic status) was noted to have increased from 139 in 2013 to 445 [17]. No previous studies on ICDM model fidelity are available. However, studies that have looked at implementation of other chronic diseases management models, highlighted a high variability in the combination of components included the chronic disease models and the ways in which they are implemented [43, 48]. In our study, the overall level of implementing the chronic care model elements according to guidelines varied between 55 and 89% [48–50]. The highest (89%) level of fidelity observed was in a continuum of care programme, with 16 of the 18 components implemented according to the guidelines [48].

In our study, there was variability between health facilities on the level of fidelity in the implementation of the ICDM model, with facility re-organization component having a significantly higher level of fidelity in WR, compared to the DKK health district. In studies on the integration of services for various chronic diseases in primary healthcare practices, there was also high level of variability in the level of implementation on each of the components [49, 50]. Although the primary healthcare monthly headcount of patients was slightly higher in the participating clinics of the DKK district, the district had fewer medical officers and nurses. The lower human resource (clinical personnel) level could have contributed to the lower fidelity in the implementation of the ICDM model in the DKK health district compared to the WR. Other contextual factors that have been described as facilitators for successful implementation and sustainability of chronic care models and were not assessed in this study, include the commitment and support of the leadership, training of personnel, participants responsiveness, sufficient funding, acceptability of the intervention and collaboration with other sectors [43, 48, 51–53]. The observed variability in fidelity level across ICDM model components and health districts could indicate adaptations to the model to fit different contexts. The availability of infrastructure and resources, the capacity of the implementing teams and time constraints are some of the factors that could lead to spontaneous adaptations of an intervention to enhance its suitability to context [29, 30]. Low fidelity in the implementation, especially if the core components have been removed, could affect the effectiveness of the intervention [30]. The impact of contextual factors on the variability in the implementation fidelity of the various activities of the ICDM model in the two health districts and how this variability affects ICDM programme effectiveness needs further research. In addition, that data would inform the implementation of the ICDM model in other health systems or populations (scaling-out).

The ICDM model activities that had low (< 70%) fidelity in our study included administration (pre-retrieval of medical records and different streams of care), health promotion and clinical supportive management by the DCST. Improvements in clinical outcomes and operations have been documented in chronic care models that provide decision support and delivery system design [43]. Lack of clinical leadership could adversely affect the expected outcomes and sustainability of the ICDM model [32, 38]. Redesign of service delivery, integration of services and decision support were also inadequately implemented in other chronic care models evaluations, with scores of 39–46%; 46 and 58% respectively [49, 50]. Clinical management decision support should be enhanced in this setting where nurses’ knowledge on chronic diseases is inadequate and guidelines are not readily available [54–57]. Although there was a high level of integration (time, healthcare provider, space) TB patients had a separate stream from patients with other chronic conditions, despite the recommendations by WHO and UNAIDS to integrate TB and HIV services [58, 59].

A high level of fidelity was discovered on integration of services and the facilitated self-management and community support with WBOTs and CHCW. Assisted self-management support was also the most prominent component of several chronic care models and resulted in improvements in health outcomes [43], and in an evaluation of other primary healthcare practices on the level of implementation on the chronic care model components, self-management support scored 48% [50]**.** Contextual adaptations (modifying the adaptable while maintaining key components of interventions) may be needed to enhance feasibility, reach, and acceptability [19, 20, 29, 30]. The ICDM model guidelines do not, but should clearly outline which are the adaptable and which key components of the model to optimise implementation fidelity, and facilitate scale-out, scale-up and process evaluations.

Regarding waiting times, 3 hours is the maximum time patients are expected to spend in a health facility when accessing services, based on the ideal clinic standards in South Africa [18]. In this study, 70% of observed patients were at the PHC facilities for 3 h or more. The high median waiting time in our study was similar to the findings of Egbujie et al., which showed that some clinics in South Africa have reduced while others have increased waiting time after the implementation of the ICRM programme [60]. Observed inefficiencies in our study included excessive waiting time and nurses’ motion and rework. There were also unintended consequences like poor adherence to guidelines on medication management in consulting rooms and redundancy of clinical staff when allocated to non-clinical ICDM model activities. The ICDM model and ICRM programme also follow the lean thinking principles on waste reduction like waiting time, excess inventory, underutilized staff and excess people motion [7, 18]. Our study found that the participating PHC facilities did not perform well on waste reduction according to these lean principles .

### Strengths and limitations

This study has a number of strengths. Firstly, multiple visits to health facilities over 8 months to observe the level of fidelity in the implementation of the ICDM model allowed us to assess clinics when they had different patient and personnel numbers. Secondly, the use of implementation research principles implies that this research ensures evidence-based decisions on ICDM model implementation improvements and on how the lesson learned could affect scale-up and scale -out and policies. Thirdly, application of patient flow analysis identified specific areas of inefficiencies in the delivery of chronic health services stream.

Limitations of this study included that the weighting of the scores of the fidelity criteria was based on the number of activities required, and not on how critical that activity was in achieving the ICDM model objectives. Some of the items on the fidelity criteria were scored based on the data provided in the structured questionnaire by the implementing healthcare workers, and this could have introduced social desirability bias. Assessments focused significantly on the presence of systems and processes that have been recommended, and not the quality of the implementation of the components.

## Conclusion

There was a high level of fidelity of implementation of the ICDM model in the two health districts, with some variability across ICDM model scores on components and PHC facilities. The highest median scores were on the ICDM model components of facility re-organization and strengthening of support systems. Relentless and continuous monitoring and evaluation of the PHC clinics on the ICRM programme and integrated clinical services is essential to ensure that these gains are not lost. Increased focus on quality in the implementation of elements that had high levels of fidelity like facility re-organization, assisted self-management and facilitated community support could further enhance efficiencies. The ICDM model items that were described as having lower degrees of fidelity (different streams of care, administration and health promotions) indicate opportunities for improvement of the current implementation of the ICDM model and how to support normalization into routine practice of the model. More research is needed to identify the determinants of ICDM model implementation fidelity and on innovative adaptations that can improve models’ processes and its implementation at local level without affecting the intended model’s outcomes.

## Supplementary information

**Additional file 1.** Implementation Fidelity of the Integrated Chronic Disease Management Model – Assessment Tool Activities.

## Data Availability

The data on the implementation fidelity of the ICDM model is available on Figshare, via the following URL: 10.6084/m9.figshare.9339029.v1

## Abbreviations

CCMDD: Centralized chronic medication dispensing and distribution
DOH: Department of Health
HIV: Human Immune Deficiency Syndrome
HPT: Hypertension
ICDM: Integrated chronic disease management
ICRM: Ideal clinic realisation and maintenance programme
ICSM: Integrated clinical services management
MRC: Medical Research Council
PHC: Primary healthcare
TB: Tuberculosis
WHO: World Health Organization
