# Supplementary material for: Process evaluation of implementation fidelity of the integrated chronic disease management model in two districts, South Africa
Source: BMC Health Serv Res. 2019 Dec 16;19:965. doi: 10.1186/s12913-019-4785-7 (PMC6916104; doi:10.1186/s12913-019-4785-7)
Supplement: Supplementary file 1 — Additional file 1. Implementation Fidelity of the Integrated Chronic Disease Management Model – Assessment Tool Activities. [file 12913_2019_4785_MOESM1_ESM.docx]

**Implementation Fidelity of the Integrated Chronic Disease Management Model – Assessment Tool Activities**

| 1. **Facility Re-organization** | **Administration of the Facility for ICDM Implementation** | 1. **Clinical Supportive Management** | **Integration of Care** | 1. **Assisted Self-Management** | **Community Ward Based PHC Outreach teams** | 1. **Strengthening of Support Systems** | **Health Information** |
| --- | --- | --- | --- | --- | --- | --- | --- |
|  | A1.Clinic Operating Hours |  | B1. Chronic conditions included for integrated care |  | C1.Ward based PHC outreach teams |  | D1. District Health Information System |
|  | A2.Patient Flow |  | B2. Healthcare provider integration |  | C2. Composition of outreach teams |  | D2. ART Data collection for ICDM |
|  | A3.Chronic patient scheduling system |  | B3. Time integration |  | C3. Training on monitoring of chronic patients |  | D3. TB Data collection for ICDM |
|  | A4.Chronic patient’s records management |  | B4. Space integration |  | C4. Health education campaigns |  | D4. NCD Data collection for ICDM |
|  | **Infrastructure** |  | B5. Records integration |  | C5. Support groups and adherence clubs |  | D5. Outcomes Data for ICDM |
|  | A5.State of building and cleanliness |  | **Guidelines and Resources** |  | C6. Screening campaigns |  | D6. Data collection tools |
|  | A6.Water supply and sanitation |  | B6. PC 101 Guidelines for Chronic Patients |  | **Community Healthcare Workers (CHW)** |  | **Medicine Supply and Management** |
|  | A7.Medical waste management |  | B7. NIMART Guidelines |  | C7. Facility dedicated CHW |  | D7. Stock card management |
|  | A8.Space for consultation |  | B8. PALSA Plus Guidelines |  | C8. CHW trained |  | D8. Re-order levels |
|  | A9.Waiting area for patients with a chronic disease |  | B9. NCD management Guidelines |  | C9. Frequency of visits to households |  | D9. Medication storage room |
|  | A10. Vital signs stations for chronic patients |  | B10. TB Treatment Guidelines |  | C10. Secondary health promotion |  | D10. Condition of dispensary or medication storage room |
|  | **Human Resource** |  | B11. ICDM Manual |  | C11. Basic point-of-care testing |  | D11. Storage areas within a dispensary or medication storage room |
|  | A11. Clinical Personnel trained on ICDM |  | **Facility ICDM Champion and ICM reporting** |  | C12. Referral of unwell patients |  | D12. Access management for a dispensary or medication storage room |
|  | A12. Clinical Personnel allocated to ICDM |  | B12. Facility ICDM Champion Identified |  | **Health Promotion** |  | **Equipment Supply and Management** |
|  | A13. Nurses trained on PC 101 Guidelines |  | B13. Champion for ICDM still functioning |  | C13. Personnel allocated to health promotion |  | D13. Essential equipment for reception |
|  | A14. Nurses trained on NIMART |  | B14. Monitoring and evaluation of ICDM implementation |  | C14. Frequency of health promotion activities |  | D14. Essential equipment for vital signs station |
|  | A15. Nurses scheduling for ICDM |  | B15. Reporting on ICDM implementation |  | C15. Place of educational talks |  | D15. Essential equipment for consulting rooms |
|  | A16. Other support staff for ICDM |  | **District Clinical Specialist Team (DCST)** |  | C16. Material on TB disease |  | D16. Essential equipment for resuscitation room |
|  | **Dispensing of Medication** |  | B16. Facility access to a District clinical specialist team |  | C17. Material on lifestyle diseases |  | D17. Equipment management |
|  | A17.Chronic medication stock at facility |  | B17. Composition of the District clinical specialist team |  | C18. Material on HIV |  | **Human Resources and Partnerships** |
|  | A18. Pre-dispensing and packaging |  | B18. Frequency of visits by the District clinical specialist team |  | **Patient down referrals** |  | D18. Integrated school health team |
|  | A19. Storage of pre-packed medication |  | B19. Clinical audits by the District clinical specialist team |  | C19. Practice of down referrals |  | D19. Support groups and Adherence clubs |
|  | A20. Stock-outs of chronic medication |  | B20. Mentoring of facility staff by the District clinical specialist team |  | C20. Criteria for down referral |  | D20. Provincial and district level leadership and support for ICDM |
|  | A21. Usage of stock cards |  | B21. Strengthening of the referral mechanisms between facility and hospitals |  | C21. Patients down referred |  | D21. M-health |
|  | A22. Supply of medication provided to chronic patients |  |  |  | C22. Level of utilization of down referrals |  | D22. Externally Funded partners’ |
|  |  |  |  |  | C23. Places for down referrals in the community |  |  |
|  |  |  |  |  | C24. CHW allocated to patients that have been down referred |  |  |
